# Analyzing the expression of the transcriptome in adipose tissue of fat- and thin-tailed sheep

**DOI:** 10.1016/j.vas.2024.100387

**Published:** 2024-08-12

**Authors:** Sana Farhadi, Karim Hasanpur, Jalil Shodja Ghias, Valiollah Palangi, Maximilian Lackner

**Affiliations:** a Department of Animal Science, Faculty of Agriculture, University of Tabriz, Tabriz 51666-16471, Iran; b Department of Animal Science, Faculty of Agriculture, Ege University, 35100 Izmir, Türkiye; c Department of Industrial Engineering, University of Applied Sciences Technikum Wien, Hoechstaedtplatz 6, 1200 Vienna, Austria

**Keywords:** Fat deposition, Fat-tail, Gene ontology, RNA-seq, Gene expression

## Abstract

Significant efforts have been made to understand how fat deposition in sheep tail is regulated in genetic, transcriptomic, physiologic, biochemical, and metabolic levels in order to elucidate the complex mechanisms underlying the energy storage, lipid metabolism in adipose tissue, adaptability to harsh environments, and evolutionary domestication. Through RNA-seq data analysis, we are able to compare the gene expression of fat-tailed sheep versus thin-tailed sheep breeds in an acceptable resolution at transcriptome level. The purpose of this study was to compare the transcriptomes of Ghezel (fat-tailed) and Zel (thin-tailed) sheep. Total RNA from subcutaneous and tail tissue samples from healthy lambs was sequenced (150b PE) to identify differentially expressed genes (DEGs) between the two mentioned tissues and between the Ghezel and Zel sheep breeds. Further downstream pathway and network analyses were conducted afterwards. The results uncovered the association of the most important DEGs such as *CAV1, ALB*, and *SOCS3* with cellular signaling pathways of lipids metabolism. It seems that the *SOCS3* gene plays an important role in the differential deposition of lipid in the tails of two phenotypically different sheep breeds. Although the detail of gene expression in the tail and subcutaneous tissues of two morphologically different breeds was decoded here, to fully understand how differential expression of the *SOCS3* gene affects the fat synthesis, further studies are needed.

## 1 Introduction

Mammals utilize the tail organ for many purposes, including communication, balance over movements, and attacking signals during mating competitions. Previously, the genomic perspective of the phenotypic difference in sheep tail fat deposition has been well documented in the broad literature. archeological evidence suggests that during domestication, thin-tailed sheep breeds were the earliest wild ancestors of the fat-tailed sheep breeds Ryder (1983). Statistically, up to 25 % of the sheep population worldwide is fat-tailed Farid, Izedifard, Edris & Makarechian (1983). Adipose tissue is a major storage location for surplus energy (Bouchard, Després & Mauriège, 1993) and tail and subcutaneous fat are the major fat storage sites in domestic animals Cheng et al. (2016). However, several theories have been proposed about the variation in adiposity volume and lipid formation and structure in sheep breeds. These theories focus on non-genetic factors such as diet and others on genetic factors Davidson (2014);Cheng et al. (2016).

Data from several studies suggest that the fat tail organ became essential for tolerance to extremely cold environmental conditions in response to global climate changes (Bouchard et al., 1993), Adaptation to nutrient-poor diets, energy balance in extremely hot seasons, influencing carcass fat content distribution, mutton quality, feed and energy consumption and costs, meat production efficiency and economic value. Currently, the morphology of fat-tailed sheep is considered an unfavorable trait in modern intensive and semi-intensive breeding systems and in relation to the marketing perspective for many logical reasons: reducing ram mating ability, and animal welfare. Added to this are the preferences of producers, dietary habits, and health concepts to avoid obesity in modern human society Nejati-Javaremi, Izadi, Rahmati, Moradi & Izadi (2007);Moradi, Nejati-Javaremi, Moradi-Shahrbabak, Dodds & Mcewan (2012).

Numerous studies have aimed to search for several potential candidate genes that control lipid metabolism in sheep, which would greatly accelerate breeding practices for thin-tailed sheep. Several recent studies of tail lipid metabolism in different sheep breeds have been performed, and a number of authors have acknowledged the RNA-seq-based evidence associated with subcutaneous adipose tissue in both Chinese small-tailed Han sheep and Dorset sheep (Miao & Luo, 2013) Some authors have promoted the advancement of diversity in the comparative differential gene expression profiles of tail adipose tissue between both fat-tailed Kazakh sheep and short-tailed Tibetan sheep (Wang et al., 2014), According to research, Guangling Large-Tailed and Small-Tailed Han sheep have transcriptomes of three adipose tissues Li et al. (2018). A comparative transcriptome analysis of Iranian sheep breeds with fat tails (Lori-Bakhtiari) and thin tails (Zel) was also presented in the literature review Bakhtiarizadeh, Salehi, Alamouti, Abdollahi-Arpanahi & Salami (2019). Our previous studies examined tail fat tissue RNA-seq profiles in native Ghezel and Zel breeds Farhadi, Shodja Ghias, Hasanpur, Mohammadi & Ebrahimie (2021). Moreover, our research meta-analysis of several studies revealed three important meta-genes involved in fat deposition Farhadi et al. (2023).

In this study, native Ghezel (fat-tailed) and Zel (thin-tailed) sheep (*Ovis aries*) are two breeds native to Iran that exhibit two extreme tail appearances as a result of both selective breeding and domestication processes (Valizadeh, 2010). The Zel sheep breed is an Iranian thin-tailed sheep breed originating from the Caspian Sea and contributing about 3 % to the Iranian sheep population Vatankhah, Moradi-Sharbabak, Nejati-Javaremi, Miraei-Ashtiani, & Vaez-Torshizi (2006); Kamalzadeh, Rajabbaigy & Kiasat (2008). Ghezel sheep, in the opposition group, with a large, fatty tail, is a fat-tailed breed geographically distributed in northwestern Iran, accounting for nearly 4 % of Iran sheep population Nabavi, Alijani, Taghizadeh, Rafat & Bohlouli (2014). Both breeds have different fat distributions in their bodies. While subcutaneous tissue is the main site for fat storage in the Zel breed, the Ghezel breed has a heavy tail for fat storage. Although the study of the genomic region associated with fat deposition in sheep has been extensively explored, few studies have focused on comparative transcriptome profiling between two native Ghezel and Zel sheep. Additional investigation of the identified potential candidate genes derived from RNA-Seq analysis of differential gene expression would improve our understanding of fat deposition genomics in these studied breeds. With this motivation, the goal of this study is to investigate comprehensive transcriptome profiling between two Iranian Ghezel and Zel sheep breeds.

## 2 Materials and methods

### 2.1 Ethics statement

Our experiment followed and signed the main ethical rules of the University of Tabriz's Research Council during all phases of the experiment, including animal rearing, slaughter and blood collection, (Protocol No. 20,170,415/39/ 44).

### 2.2 Animals and samples

Four tissue sample from two healthy lambs were used for the current study. One of the lambs was from fat-tailed Ghezel and another was from thin-tailed Zel breed. Both lambs experienced similar rearing conditions and were fed the same diet for 120 days. They were slaughtered at the Research Station (Khalatpoushan) of the Faculty of Agriculture, University of Tabriz, Tabriz, Iran. Adipose tissue samples were carefully harvested from the tail and subcutaneous tissues of each lamb. The samples were immediately frozen in liquid nitrogen and kept at −80 °C until RNA isolation.

### 2.3 RNA **extraction** and RNA sequencing

Total RNA was isolated from the tail and subcutaneous adipose tissue, separately, using Trizol reagent (TaKaRa, USA) according to the manufacturer's instructions. Later, the integrity and concentration of the isolated RNA were assessed using the 2100 Bioanalyzer (Agilent Technologies, Waldronn, Germany). Finally, RNA samples with a 28 s/18 s ratio > 1, an OD_260_ nm /OD_280_ ratio > 1.9, and RNA integrity (RIN) number > 7.0 were chosen for RNA sequencing. Four cDNA libraries (two per each lamb) were generated which were sequenced with the Illumina HiSeq2000 platform (150b paired-end). The generated RNA-Seq raw data was imported into the NCBI SRA database with accession number PRJNA602392 BioProject.

### 2.4 Quality control, mapping and quantification

FastQC (v0.11.5) (Andrews, 2010) and Trimmomatic (v0.35) (Bolger, Lohse & Usadel, 2014) software were used for quality control and trimming/filtering of raw sequencing reads, respectively. Raw reads with adapter contamination and more than 10% of unknown bases as well as with more than 50% of low-quality bases were trimmed out. The clean reads were mapped to the Ensembl sheep reference genome (V3.0) (ftp://ftp.ncbi.nlm.nih.gov/genomes/Ovis_aries/) utilizing Bowtie2 (v2.3.4) (Langmead & Salzberg, 2012), SAMtools (v1.3.1) (Li et al., 2009) and TopHat (v2.1.1) (Trapnell, Pachter & Salzberg, 2009) software. Assembled reads were annotated with the NCBI reference annotation by Cufflinks (v2.2.1) (ftp://ftp.ncbi.nlm.nih.gov/genomes/Ovis_aries) Trapnell et al. (2012). The individual transcripts were merged into a single transcript using Cuffmerge Mathworks (2024).

### 2.5 Gene expression analysis

Mapped read count values were normalized for both gene length and library size using FPKM (Fragments Per Kilo based on Exon Per Million Fragments Mapped) criteria Mortazavi, Williams, Mccue, Schaeffer, & Wold (2008). Gene expression profiles of both tail and subcutaneous tissues were compared between the Ghezel and Zel breeds. The differentially expressed genes between the fat- and thin-tailed samples were identified using Cuffdiff. In addition, regardless of breed origination, the gene expression profile of the subcutaneous tissue was compared with that of the tail tissue. Genes with a log2-fold change > 1.1 and a q-value < 0.05 were considered as differentially expressed which will be called as DEGs, hereafter.

### 2.6 Gene ontology classification and KEGG pathway analyses

In order to analyze GO enrichment for DEGs, we used a web-based tool. Utilizing the Enrichr database, functional enrichment analysis was conducted on the identified DEGs (https://amp.pharm.mssm.edu/Enrichr) and Kyoto Encyclopedia of Genes and Genomes (KEGG) pathway analysis (http://www.genome.jp. kegg/). GO terms or KEGG pathways with p-value < 0.05 were assumed to be significantly enriched.

### 2.7 Protein-Protein interaction network and module analysis

The DEGs of each comparison were entered separately into the STRING database. To construct the protein-protein interaction networks, all DEGs were imported into the STRING database (v 11.0) (https://string-db.org/) (Szklarczyk et al., 2016) based on experimentally validated gene fusion databases, co-expression databases, and neighborhood interactions. An interaction confidence value < 0.4 was taken into account. To identify the functional modules, the generated networks were clustered into the two modules using the K-Means algorithm. Additionally, the Cytoscape plugin cytoHubba (v 3.7.2) was used to detect the Hub genes via the Maximum Clique Centrality (MCC) method Chin et al. (2014).

### 2.8 Validation of data

To confirm the accuracy of the DEGs identification, another similar RNA-Seq dataset was retrieved from the GEO database (PRJNA508203) and analyzed in the same way to provide validation results.

## 3 Results

### 3.1 Sequencing data and mapping summary

Almost 86 % of clean reads were successfully mapped to the sheep genome sequence (*Ovis aries*). Table 1 displays summarizes the mapping characteristics of the data of subcutaneous and tail tissues of the two breeds studied.

**Table 1:** Summary of the analyzed data and its mapping metrics.

Read	Subcutaneous		Tail	
	Zel	Ghezel	Zel	Ghezel
Total reads	50,865,488	50,533,620	43,330,598	43,767,392
Total base pairs	7629,823,200	7580,043,000	6499,589,700	6565,108,800
Total mapped reads	46,057,524	45,994,640	34,616,084	35,112,150
Total mapped reads%	(87.8%)	(83.3%)	(86.7%)	(86.8%)
Multi-mapped reads	2532,532	2678,096	2297,504	2371,892
Unmapped reads	2275,432	1860,884	8714,514	8655,242

### 3.2 Identification of differentially expressed genes in ghezel and zel sheep breeds

Differential analysis showed a total of 25, 11, 23, and 16 DEGs between subcutaneous of Zel *vs.* Ghezel, tail of Zel *vs*. Ghezel, subcutaneous *vs.* tail within Zel, and subcutaneous *vs.* tail within Ghezel, respectively. For each comparison, the top up- and down-regulated genes are presented in Table 2. As can be seen in Table 1, almost all of the DEGs are closely related to lipid metabolism. The complete list of DEGs is available in the supplementary spreadsheet file (S1).

**Table 2:** Top up- and down-regulated differentially expressed genes (DEGs) in four comparisons.

Comparison	Up-regulated	Log_2_ (FC)	Down-regulated	Log_2_ (FC)
Zel-subcutaneous *vs*. Ghezel-subcutaneous	ENSOARG00000021831	+6.56	ALB	−5.51
Zel-tail *vs*. Ghezel-tail	CAV1	+3.37	ENSOARG00000012750	−4.03
Zel -subcutaneous *vs.* Zel-tail	SOCS3	+4.30	S100A8	−3.46
Ghezel -subcutaneous *vs*. Ghezel-tail	BMP5	+4.29	ENSOARG00000021831	−5.36

FC: foldchange.

### 3.3 Functional enrichment analysis

**Fig. 1:**
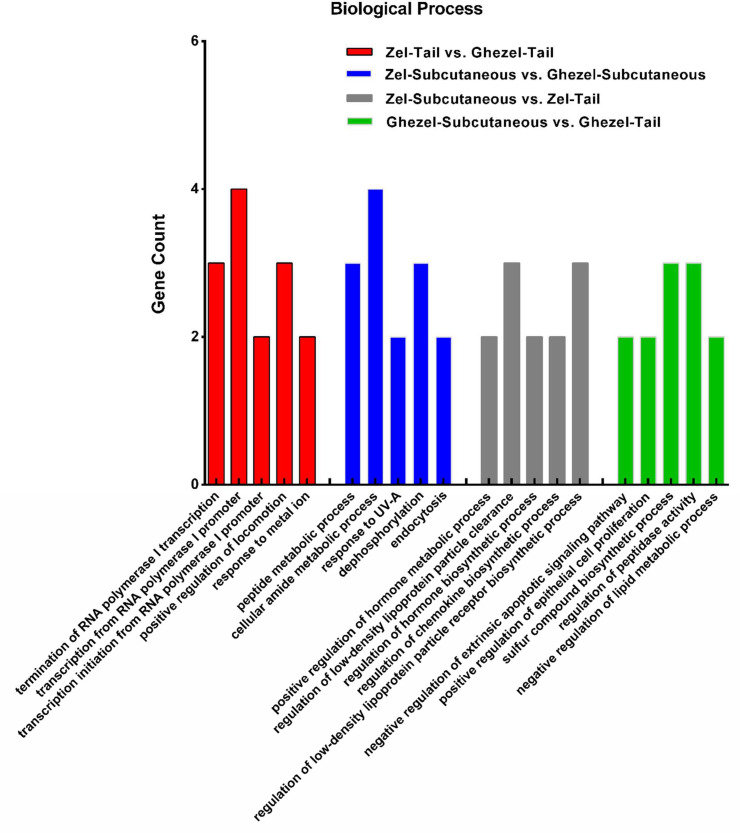
Gene Ontology analyzes of differentially expressed genes reveal the top 5 biological process terms for each comparison. Some of that are related to fat metabolism.

A Gene Ontology enrichment analysis shows that the DEGs of all comparisons were significantly enriched in terms of cellular component term "mitochondria" and molecular function term "DNA binding" (p-value<0.05). In Fig. 1, top 5 biological process (BP) terms for each comparison are shown.

Enrichment analysis showed that the DEGs of Zel-Subcutaneous *vs*. Ghezel-Subcutaneous, Zel-Tail *vs*. Ghezel-Tail, Zel-Subcutaneous *vs.* Zel-Tail and Ghezel-Subcutaneous *vs*, Ghezel-Tail comparisons were significantly enriched in 6 (72), 12 (15), 57 (49) and 12 (82) biological process terms and KEGG (Kyoto Encyclopedia of Genes and Genomes) pathways, respectively. Most of the terms were associated with fat metabolism such as “peptide metabolic process”, “negative regulation of lipid metabolic process”, “regulation of peptidase activity”, “negative regulation of steroid biosynthetic process”, “negative regulation of lipid biosynthetic process” and “regulation of low-density lipoprotein particle receptor”. Pathway analysis showed similar patterns; for instance, “Adipocytokine signaling pathway”, “TNF signaling pathway”, “Steroid biosynthesis”, “Renin-angiotensin system” and “Glycolysis/Gluconeogenesis”. In Table 3, top five significantly enriched KEGG pathways of all four comparisons are illustrated.

**Table 3:** Top five significantly enriched KEGG pathways of all four comparisons.

Comparison	KEGG Terms	Enriched Genes	P-value
Subcutaneous of Zel *vs.* Ghezel	Steroid biosynthesis	*GSTA1, TNNC1, DUSP26, CCDC69, ALB, GSTA1*	0.0023
	Dilated cardiomyopathy (DCM)	*GSTA1, TNNC1, DUSP26, ENSOARG00000026007*	0.0024
	Hypertrophic cardiomyopathy (HCM)	*CCDC69, ENSOARG00000026007, ENSOARG00000011304*	0.0024
	Cardiac muscle contraction	*SNCB, CCDC69, IFI6, GSTA1*	0.0024
	Metabolic pathways	*IFI6, SNCB, CCDC69,*	0.0024
Tail of Zel *vs*. Ghezel	AGE-RAGE signaling pathway in diabetic complications	*TNNC1, CAV1, ENSOARG00000012750, MYO18B*	0.0006
	Osteoclast differentiation	*OTOR, SCTR, HOXC12 S100A8*	0.0006
	Rheumatoid arthritis	*MME, MYO18B,*	0.0123
	Non-alcoholic fatty liver disease (NAFLD)	*S100A8, CAV1*	0.0123
	Insulin resistance	*TNNC1, KLHDC8B*	0.0123
Subcutaneous *vs.* Tail within Zel	Renal cell carcinoma	*ENSOARG00000015390, S100A8, ENSOARG00000000895*	0.0003
	HIF-1 signaling pathway	*TNNC1, CAV1, KLHDC8B, SOCS3*	0.0006
	IL-17 signaling pathway	*FMOD, BMP5, SOCS3*	0.0008
	TNF signaling pathway	*S100A8, ENSOARG00000017609, SOCS3*	0.0069
	Adipocytokine signaling pathway	*CA5A, ENSOARG00000000895, BMP5*	0.0069
Subcutaneous *vs*. Tail within Ghezel	Jak-STAT signaling pathway	*EDIL3, ENSOARG00000026007, CTBS, SOCS3*	0.0002
	Th17 cell differentiation	*ENSOARG00000001701, CA5A, ENSOARG00000024837*	0.0004
	Adipocytokine signaling pathway	*SOCS3, ENSOARG00000005855, ANXA5*	0.0004
	Prolactin signaling pathway	*ENSOARG00000024837, ENSOARG00000026007*	0.0005
	Th1 and Th2 cell differentiation	*CTBS, RF00017*	0.0006

### 3.4 Protein–Protein interaction (PPI) network and module analysis

**Fig. 2:**
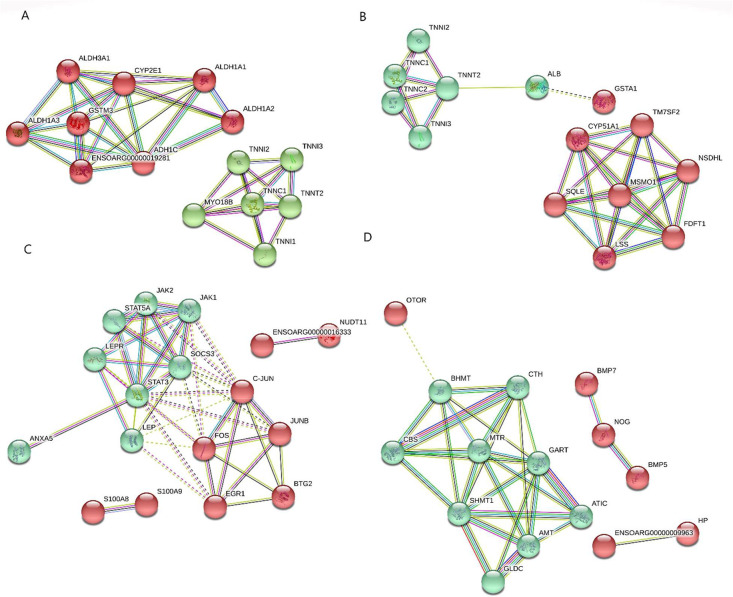
Analysis of PPI network and functional modules Zel-Tail *vs*. Ghezel-Tail (A) Zel- Subcutaneous *vs*. Ghezel-Subcutaneous (B), Zel-Subcutaneous *vs*. Zel-Tail (C) and Ghezel-Subcutaneous *vs*. Ghezel-Tail (D) comparisons. *SOCS3* as a hub gene among the DEGs of subcutaneous *vs*. tail tissues within Zel (C).

The results showed that the PPI networks were significantly enriched by the DEGs (p-value < 0.05). *LOC101107401*, cytochrome P450 family 51 subfamily A member 1 (*CYP51A1*), suppressor of cytokine signaling 3 (*SOCS3*), and cystathionine synthase (*CBS*) were identified as hub genes from the PPI networks that were constructed by the DEGs of tail of Zel *vs*. Ghezel, subcutaneous of Zel *vs*. Ghezel, subcutaneous *vs*. tail within Zel, and subcutaneous *vs*. tail within Ghezel, respectively. In Fig. 2 the PPI network and functional modules of the four comparisons are shown.

### 3.5 Validation of the differentially expressed genes

**Fig. 3:**
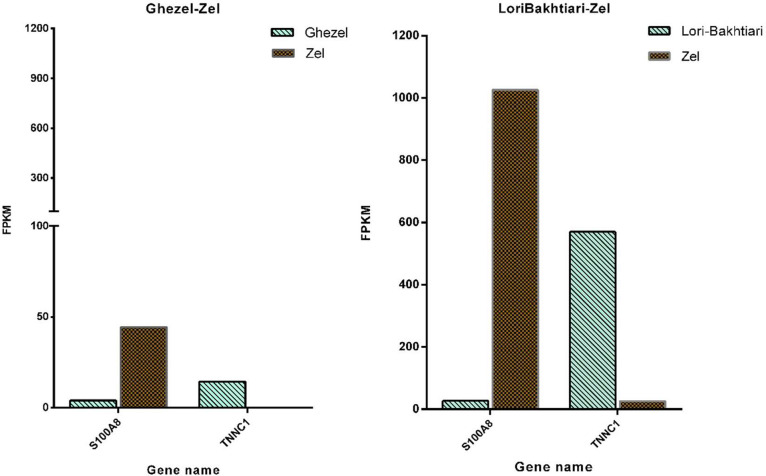
Gene expression two differentially expressed genes (DEGs) in the current study and in the study by Bakhtiarizadeh et al. (2019).

The DEGs of the current study were validated by reanalyzing the data from the study by Bakhtiarizadeh et al. (2019). There were four common DEGs including *MYO18B, TNNC1*, S100A8 and *DUSP26* between the DEGs of tail of Zel *vs*. Ghezel breeds in the current study and the mentioned study. A similar expression pattern had been shown by Bakhtiarizadeh et al. (2019) in their comparison of q-RT-PCR with RNA-seq data for two out of the four mentioned genes (i.e., *TNNC1*, S100A8). We compared the expression pattern of the two mentioned genes in our study with that of Bakhtiarizadeh et al. (2019). In Fig. 3, the expression pattern of two DEGs of the current work (tail of Zel *vs*. Ghezel breeds) has been compared with that of the Bakhtiarizadeh et al. (2019) study.

## 4 Discussion

There are hundreds of sheep breeds, worldwide. While the majority of the world's sheep population originate from the thin-tailed sheep breeds, almost one-fourth of them originate from the fat-tailed sheep breeds. The fat-tailed sheep breeds are mainly distributed in arid and desert-like areas in Middle-East, northern parts of Africa, and central parts of Asia. Iran, one of the Middle-East countries, possessing almost 50 million sheep from 28 breeds, is well known for its richness in sheep breeds which vary in multiple phenotypic characteristics from the very heavy body and tail (such as Ghezel which was originally distributed in northwest portion of Iran) to a small thin-tailed sheep breed named Zel. It is mainly distributed in north and rainy areas of Iran around the Caspian Sea coast. The shape and size of the tail may reflect the sheep's history of evolution in numerous arid and semi-arid parts of Iran. In other words, during domestication, there has been intense selection pressure for the genes responsible for the creation of fat-tailed breeds, either naturally or artificially. Nevertheless, the identification of genes responsible for heavy fat-tail, which could be possible via the comparative whole transcriptome study of the fat-tailed breeds versus the thin-tailed breeds, might be important from both genetic and evolutionary points of view. In this study, we investigated the pattern and variability of transcriptome profile of tail and subcutaneous tissues between the Ghezel and Zel sheep breeds. We hypothesized that the different patterns or profiles of gene expression between the two mentioned breeds may be beneficial in detecting the most significant genes responsible for the heavy, fat tail. The study resulted in a surprisingly low number of significant DEGs with 25, 11, 23, and 16 genes differentially expressed between the subcutaneous of Zel *vs*. Ghezel, tail of Zel *vs*. Ghezel, subcutaneous *vs.* tail within Zel, and subcutaneous *vs.* tail within Ghezel, respectively. Besides, the rearing protocol of the used lambs as well as their gender and age were completely similar. We found little consistency among the four DEG lists as different sets of DEGs were identified in the four comparisons indicating that the expression profiles of the tail and subcutaneous fats are significantly different between the two breeds. It was not surprising as the morphology of fat was considerably different between the two tissues. In addition, the sampling from the tail and subcutaneous adipose tissues was performed at later stage of adipocyte differentiation. Therefore, a high level of expression of these genes in fat-tailed adipocytes indicated their importance for adiposity metabolism. According to Table 1, one of the important genes in the synthesis of fatty acids is Caveolae Associated Protein 1 (*CAV1*). Knocking down the *CAV1* gene has resulted in light fat weight and reduced white fat deposits Ding et al. (2014). In addition, the importance of the *CAV1* gene for hepatic lipid homeostasis and nuclear hormone receptor (PPARa) and bile acid signaling has been highlighted earlier Astudillo et al. (2011). Therefore, these results suggest that *CAV1* deficiency impairs PPARa signaling in metabolically active tissues such as liver and white adipose independent of fatty acid availability Kadowaki et al. (2003). Overexpression of *CAV1* gene may play a strong role in significantly more lipogenesis of fatty acids in the tail of Ghezel breed. Furthermore, lower expression of some lipolysis-related genes (such as *SOCS3*) in Ghezel than Zel may indicate either the inhibition of lipolysis in Ghezel or the promotion of lipolysis in Zel. Recent research has shown that the *SOCS3* gene plays an important role in regulating fatty acid oxidation Luo et al. (2011). Albumin (*ALB*) is also one of the important fatty acid-binding proteins in extracellular fluids. Plasma albumin has at least 7 fold higher affinity to fatty acid binding sites that increase the concentration of fatty acids. Free fatty acids are transported into cells by being conjugated to plasma albumin, which is activated using ATP to form acyl-CoA. Experimental evidence suggests that albumin facilitates the uptake of fatty acids into organs. For example, according to the study of Mackenzie et al. the ratio of albumin to fatty acid controls the lipogenic activity of blood serum Mackenzie, Mackenzie, Reiss & Wisneski (1970). Compared to subcutaneous tissue of Ghezel breed, higher expression of ALB gene in subcutaneous tissue of Zel breed may explain the fact that higher quantity of fat is deposited in subcutaneous adipose tissue of Zel breed. *CAV1, SOCS3, ALB* were three most significantly differentially expressed genes that are believed to be associated with fat storage.

Several lipolysis-related pathways, such as “Jak-STAT signaling pathway”, “IL-17 signaling pathway”, “Adipocytokine signaling pathway”, “Osteoclast differentiation” and “TNF signaling pathways” were enriched by the DEGs of subcutaneous *vs.* tail tissues within Zel breed, suggesting that lipolysis of fat in the tail of Zel sheep is much facilitated than in the subcutaneous tissue. As was mentioned above, “Adipocytokine signaling pathway” is one of the significant pathways of subcutaneous *vs*. tail comparison in Zel sheep. Adipocytokines secreted by adipose tissue, such as *IL-6* and TNF-α, play a main regulative role in the energy metabolism of cell fatty acids and regulate the fat metabolism via the activation of AMP-activated protein kinase (AMPK), which phosphorylates/inactivates acetyl-coenzyme-A carboxylase, and then decreases the production of malonyl CoA. Fatty acid synthase is regulated by malonyl CoA, which suppresses mitochondrial transport of fatty acids Kadowaki et al. (2003). This process decreases fatty acid synthesis, increases fatty acid oxidation, and decreases the triglyceride storage in adipose tissue Kadowaki et al. (2003). Therefore, adipocytokines are known as lipolytic factors that increase adipocyte lipolysis. This function of adipocytokines may explain the reason for thinner tail while heavy deposition of fat in subcutaneous tissue in Zel (thin-tailed) breeds. *SOCS3* is one of the main genes of this pathway that contribute to the regulation of β-oxidation of lipids as well as in enhancing lipolysis and decreasing the expression of genes related to lipogenesis. Besides being a hub gene in the PPI network of DEGs of subcutaneous *vs.* tail within Zel breed, *SOCS3* also enriched in several other significant KEGG pathways including “Jak-STAT signaling pathways”, “TNF signaling pathways” and “IL-17 signaling pathways” that are all related to lipid lipolysis.

The “renin-angiotensin system pathway” is another important pathway when comparing subcutaneous tissues between the Zel and Ghezel breeds, which plays a critical role in adipocyte differentiation and body fat storage. Angiotensinogen expression and secretion are increased during adipogenesis Schling, Mallow, Trindl & Löffler (1999). Elevated adipocyte angiotensinogen concentrations have been reported during the development of obesity in rats Hainault et al. (2002). In addition, an in vitro study on human subcutaneous adipocytes has reported that angiotensinogen protein expression is increased with increasing insulin concentrations Harte et al. (2003). In mice, stimulation of preadipocytes by Ang II was associated with increased expression of glycerol-3-phosphate dehydrogenase and fatty acid synthase, which are two significant markers of fat development Gregoire, Smas & Sul (1998). Membrane metallo-endopeptidase (*MME*) was one of the DEGs involved in this pathway, which can target the degradation of lipolytic factors such as *IL-6* and amyloids Ramirez et al. (2019). *IL-6* is a known lipolytic factor that increases lipolysis of adipocytes by breaking down the lipids and oxidizing the fatty acids Zhang et al. (2014). *IL-6* has been shown to stimulate the lipolysis and fat oxidation in humans (Van Hall et al., 2003), mice (Ma, Gao, Sun & Liu, 2015), rats (Nonogaki et al., 1995), bovine (Contreras et al., 2017) and sheep Farhadi et al. (2021). Amyloid also stimulates lipolysis via PKA and ERK1/2 dependent pathways and induces leptin and *IL-6* secretion. Thus, it can be concluded that amyloid peptide has a functional effect on adipose tissue and can lead to an increased release of free fatty acids Wan, Mah, Simtchouk, Kluftinger & Little (2015). These results suggest a remarkable role for renin-angiotensin system pathway in increasing fat deposition in the subcutaneous tissue of the Zel breed. Therefore, up-regulation of the *MME* gene in the subcutaneous tissue of the Zel breed could be associated with the higher deposition of fat in subcutaneous tissue than in the tail tissue.

*LOC101107401, CYP51A1, SOCS3, and CBS* genes were identified as hubs in the PPI networks constructed by the DEGs of tail of Zel *vs*. Ghezel, subcutaneous of Zel *vs*. Ghezel, subcutaneous *vs.* tail within Zel, and subcutaneous *vs.* tail within Ghezel. *CYP51A1* gene catalyzes the synthesis of cholesterol and other lipids by encoding a cytochrome P450 superfamily of enzymes Liu et al. (2015). In addition, *CYP51A1* gene plays an important role in lipid metabolism by increasing fatty acid and sterol synthesis and suppressing fatty acid oxidation. Therefore, up-regulation of *CYP51A1* gene can result in an increased fat deposition in the subcutaneous tissue than in tail of Zel sheep. *CYP51A1* gene was a member of the red module in the PPI network (Fig. 2B) that enriches steroid biosynthetic pathways. Conversely, the *CBS* hub gene is involved in the regulation of triglycerides, cholesterol and lipogenic enzymes via the lipogenic transcription factors SREBP1 and SREBP2. There have been reports that knockdown of the *CBS* gene results in decreased expression of key enzymes involved in lipid synthesis such as *FASN* and *ACC1* Chakraborty et al. (2015). Furthermore, up-regulation of the *CBS* gene in the tail of Ghezel may accelerate the deposition of lipids in the adipocytes due to the *CBS* role in lipogenesis. *CBS* gene was a member of the green module in the PPI network (Fig. 2D) that enriches the metabolism of glycine, serine, and threonine, as well as the metabolic pathways of cysteine ​​and methionine, which could indicate the nature of diversity between sheep breeds. This finding suggests that up-regulation of these genes may result in an increased fat deposition in the tail fat of the Ghezel breed in comparison to that of the both Ghezel and Zel subcutaneous tissues.

## 5 Conclusion

Based on the findings of the current work we identified some DEGs that were in close association with lipid metabolism. Our results show that some lipid-related pathways such as "Jak-STAT signaling", "IL-17 signaling", "adipocytokine signaling", "osteoclast differentiation" and "TNF signaling" may be enhanced lipolysis of adipose tissues in Zel breed. Also, some metabolic pathways such as "steroid biosynthesis", "renin-angiotensin system" and "glutathione metabolism" may be involved in fat lipogenesis tissue in Ghezel breed. Some genes related to lipid metabolism, such as *MME, CANIN1, ALB*, and especially *SOCS3*, may be genetic factors responsible for the variability in the rate and amount of fat deposition between the Ghezel and Zel sheep breeds. We also identified *SOCS3* as a hub gene among the DEGs of subcutaneous *vs*. tail tissues within Zel breed. Overall, our results suggest a strong effect of the *SOCS3* gene on lipid metabolism in thin-tailed sheep breeds. To the best of our knowledge, this is a novel finding in comparative transcriptome changes associated with Iranian fat- and thin-tailed sheep breeds. Future studies could investigate this issue further by including more breeds and a larger sample size in the experiment. This would be among two extreme classes of fat- and thin-tailed sheep breeds.

## Informed consent statement

Informed consent was obtained from all subjects involved in this study.

## CRediT authorship contribution statement

**Sana Farhadi:** Writing – original draft. **Karim Hasanpur:** Writing – original draft. **Jalil Shodja Ghias:** Writing – original draft. **Valiollah Palangi:** Writing – review & editing. **Maximilian Lackner:** Writing – review & editing.

## Declaration of competing interest

The authors declare that they have no known competing financial interests or personal relationships that could have appeared to influence the work reported in this paper.

## Appendix Supplementary materials

Image, application 1
