# Supplementary material for: Analyzing the expression of the transcriptome in adipose tissue of fat- and thin-tailed sheep
Source: Vet Anim Sci. 2024 Aug 12;25:100387. doi: 10.1016/j.vas.2024.100387 (PMC11381445; doi:10.1016/j.vas.2024.100387)
Supplement: Supplementary file 1 [file mmc1.docx]

**Total differentially expressed genes (DEGs) in four comparisons**

| Subcutaneous of Zel *vs.* Ghezel | Tail of Zel *vs*. Ghezel | Subcutaneous *vs*. tail within Ghezel | Subcutaneous *vs.* tail within Zel |
| --- | --- | --- | --- |
| *CA5A* | *ENSOARG00000015390* | *ENSOARG00000024837* | *SP9* |
| *ENSOARG00000021831* | *CAV1* | *SCTR* | *NUDT11* |
| *ENSOARG00000026007* | *ENSOARG00000019281* | *HOXC12* | *ENSOARG00000026988* |
| *CLDN4* | *DUSP26* | *ENSOARG00000000895* | *S100A8* |
| *CPXM2* | *S100A8* | *CTBS* | *ENSOARG00000016333* |
| *GSTA1* | *TNNC1* | *BMP5* | *ENSOARG00000017609* |
| *DUSP26* | *SNCB* | *EDIL3* | *ENSOARG00000001279* |
| *TRPM2* | *ENSOARG00000012750* | *ENSOARG00000001701* | *SOCS3* |
| *RF00017* | *KLHDC8B* | *CBS* | *EGR1* |
| *RF00100* | *MYO18B* | *RF00030* | *HNRNPK* |
| *RF00017* | *ENSOARG00000017609* | *CA5A* | *CAVIN1* |
| *IFI6* |  | *OTOR* | *BTG2* |
| *TNNC1* |  | *ENSOARG00000025779* | *ENSOARG00000005855* |
| *SCTR* |  | *ENSOARG00000021831* | *ENSOARG00000026272* |
| *ENSOARG00000011304* |  | *RF00017* | *ANXA5* |
| *TMEM72* |  | *FMOD* | *ENSOARG00000025516* |
| *SNCB* |  | *ENSOARG00000026007* |  |
| *ALB* |  | *ENSOARG00000009963* |  |
| *CCDC69* |  | *APOD* |  |
| *CYP51A1* |  | *CLDN4* |  |
| *ENSOARG00000009277* |  | *ENSOARG00000003744* |  |
| *VLDLR* |  | *ECM1* |  |
| *MME* |  | *IFI6* |  |
| *PTPN4* |  |  |  |
| *KLHDC8B* |  |  |  |
